# A survey of nursing teachers’ awareness of discrimination and inequity in telephone nursing care

**DOI:** 10.1186/s12912-021-00762-5

**Published:** 2021-12-02

**Authors:** Inger K Holmström, Elenor Kaminsky, Anna T Höglund, Marianne Carlsson

**Affiliations:** 1 School of Health, Care, and Social Welfare, Mälardalen University, Box 883, SE- 721 23 Västerås, Sweden; 2 Department of Public Health and Caring Sciences, Uppsala University, Box 564, SE-751 22 Uppsala, Sweden; 3 Faculty of Health and Occupational Studies, University of Gävle, SE-80176 Gävle, Sweden

**Keywords:** Discrimination, Equity, Telephone nursing, Nursing teachers

## Abstract

**Background:**

Nursing care should be respectful of and unrestricted by patients’ age, ethnicity, gender, dis/abilities or social status, and such values should be taught to nursing students. Nursing teachers are crucial as role models, and their values are essential. In telephone nursing, only age, sex and ethnicity are known to the registered nurses, which can be challenging. The aim of this study was to explore awareness of discrimination and inequity in telephone nursing among nursing teachers.

**Methods:**

A study specific survey was filled in by 135 nursing teachers from three universities in Sweden. The survey included short descriptions of 12 fictive persons who differed in age, ethnicity and sex and with questions about their estimated life situation. The teachers’ estimations of life situation were ranked from lowest probability to highest probability. A ‘good life index’ was constructed and calculated for each fictive person. It included quality of life, power over one’s own life and experience of discrimination.

**Results:**

The results indicate that the nursing teachers were aware of how power and age, ethnicity and sex are related; that is, they were aware of discrimination and inequity in healthcare. The persons assessed to be most likely to lead a good life were males of Swedish origin, followed by females of Swedish origin. Persons with non-European origin were estimated to have the highest probability of experiencing discrimination.

**Conclusions:**

The nursing teachers were aware of discrimination and inequity in healthcare. They were able to estimate a fictive person’s life situation based on the limited knowledge of age, ethnicity and sex. This is important, as their values are pivotal in theoretical and practical nursing education.

**Supplementary Information:**

The online version contains supplementary material available at 10.1186/s12912-021-00762-5.

## Background

Equal value and rights for all humans, irrespective of their age, sex, religion and ethnicity are stipulated in the United Nations’ (UN) Universal Declaration of Human Rights [1]. Further, Article 12 of the International Convention on Economic, Social and Cultural Rights [2] states ‘the right of everyone to the enjoyment of the highest attainable standard of physical and mental health’. For registered nurses worldwide, the International Council of Nurses’ (ICN) ethical code of conduct [3] is a core guiding principle for the profession, which stipulates that, ‘Nursing care is respectful of and unrestricted by considerations of age, colour, creed, culture, disability or illness, sex, sexual orientation, nationality, politics, race or social status’ (p. 1). Equal care and good health for all citizens are accordingly established in the Swedish Healthcare Act [4] and the Patient Act [5] and has for decades been the guiding principle for healthcare. This means that teaching nursing students these values is essential in nursing education. Therefore, nursing teachers’ values and role modelling are important. Students are able to detect both positive and negative values [6].

Despite the aforementioned regulations and policies, discrimination of individuals and groups occurs in healthcare. Discrimination can be defined [7] as instances ‘when a person is treated unfavourably or when a person’s dignity is violated’. Discrimination relates to the treatment of individuals or groups in a way that refrains from the principle of sameness, namely that individuals who are in some respect equal should be treated equally. When individuals are treated disadvantageously, based on arbitrary or irrelevant factors such as age, sex, skin colour and so on, discrimination is at hand [8]. To favour persons of a certain sex to obtain an expensive medicine (provided that the medicine works equally well independently of sex) would be classified as discrimination. The United Nations has identified non-discrimination and equality as foundations of the rule of law [1]. *Equality* refers to the principle of sameness, in that it promotes justice through ensuring people the same rights. However, in relation to discrimination, *equity* also is important, because that term refers to fairness and ensuring everyone the same opportunities.

Within Swedish healthcare, a study of formal patient complaints revealed that experiencing discrimination, disrespect and being ignored were among the reasons for the complaints [9]. Examples of discrimination are, for instance, that men to a higher extent than women are prescribed expensive medicines and that females might have to wait longer for an ambulance or for an appointment at a general practitioner (GP) [10] or surgery [11].

One way to decrease inequity and discrimination in healthcare is by increasing nursing students’ cultural competence. Such competence has be defined as the ability of an individual or an organisation to function effectively within different cultural situations [12, 13]. However, educational efforts to increase cultural competence showed varying effects [14]. After the integration of cultural-aspect nursing in a curriculum at the University of Hawaii, students viewed themselves to be somewhat prepared to provide culturally competent care. Lack of role models was one of the causes for this limited preparedness [15].

Swedish nursing students showed a high awareness of inequity in health in a previous study [16]. They also reported high consciousness concerning the interaction between different power structures, such as age, sex and ethnicity [16]. Principles of equal care in accordance with the ICN ethical code of conduct [3] are taught in Swedish nursing education. Hence, nursing teachers have a pivotal role in framing the values of the future nursing workforce [17]. An important part of learning in clinical and academic contexts is the reframing of personal values. Also, critical reflective practice is important, to support students’ learning of ethical values [18]. Nursing education responds to both local and global demands [17]. A Turkish study showed correlations between nursing students’ professional values and a positive attitude towards older persons [19], which might ensure that they do not enact ageism. It is thus important to identify competencies [20] and values needed for nursing teachers to educate highly competent nurses, and there is a growing awareness of the need to educate nurses to be sensitive to needs of different groups in the society, such as LBGT+people [21].

The Swedish nursing teachers are either junior lecturers with a master’s degree or senior lecturers with a PhD degree. Other staff members, such as chaired professors or PhD students, are also involved in nursing education. The Swedish nursing curriculum is three years in duration, leads to a Bachelor’s degree and follows regulations from the Swedish government and the Swedish Higher Education Authority [22]. The program includes courses in communication and nursing ethics. Specialist nursing curricula, such as district nursing, include advanced training in communication, as for motivational interviewing and telephone nursing.

Telephone nursing provided by the national Swedish Healthcare Direct (SHD), operating 24 h a day, every day, 52 weeks a year, is recommended as the citizens’ first contact with healthcare. Registered nurses provide care in about 5.5 million calls/year [23]. Calls are generally short, about five minutes [24], and telephone nurses have access to limited information about the caller. Often, however, they know the caller’s name, age and legal sex. The encounter by telephone is hence faceless [25], so it is more difficult for the registered nurse to know whether the caller is embarrassed, uncomfortable or afraid [26]. According to the French philosopher Lévinas, the encounter between individuals raises a moral claim, based on the fact that we meet the other person as ‘a face’ [27]. This face-to-face encounter remains as the basis for ethics between people [27].

In addition, telephone nurses have a gatekeeping role [28], and it is hence of outmost importance for assessments to be made according to the symptom severity and not on preconceptions about age, ethnicity and sex, for example. However, previous studies have revealed that males and fathers, to a greater extent than females and mothers, are referred to or recommended for a physicians’ appointment [29, 30], and gendering was a discourse in the calls [31]. The same pattern has been shown in other countries [32]. Females are also known for presenting with more worry when calling [33].

The Swedish society is multicultural, with a proportion of 18.5% of citizens born abroad [34]. This makes studies of equity in healthcare even more important in this context. Arguable, such studies need to take into account the intersectionality of different structures, such as age, sex and ethnicity.

To sum up, laws and regulations stipulate that healthcare should be provided equally for all citizens. However, previous studies have shown that inequalities occur within healthcare, such as the use and outcome of calls to SHD [26, 29–31]. The nurse-patient encounter is likely to be affected by awareness of inequity and intersectionality perspectives. Because nursing teachers’ awareness of discrimination and inequity is likely to influence nursing students’ attitudes towards patients, these are important to explore. The present study is part of a larger project investigating aspects of intersectionality and care on equal terms in telephone nursing and educational settings.

Therefore, the aim of the present study was to explore nursing teachers’ awareness of discrimination and inequity in telephone nursing.

## Method

### Design

A descriptive design with a quantitative survey approach was used.

### Sample and setting

A convenience sample consisting of 135 nursing teachers, from three university educational settings in central Sweden, was used. The survey was distributed to 195 nursing teachers, and 135 answered the survey, a response rate of 68%. Nine of the respondents were male and 126 were female. The mean age for the sample was 51.29 years (SD = 8.83). The females were slightly older, M = 51.32 years, (standard deviation [SD] = 0.78) compared to the males, M = 50.89 years (SD = 3.68). Of the nursing teachers, 78 were junior lecturers, 51 were senior lecturers and eight had other positions.

### Procedure

A study specific paper survey was filled out by nursing teachers, either at staff meetings or at home, during August and September 2018.

### Ethical considerations

According to Swedish legislation [35], no formal approval of the study was required, because it did not deal with sensitive, personal data or risk affecting the participants’ health and well-being in any way [35]. In all steps of the work, the study adhered to the ethical principles of the Declaration of Helsinki and to the Swedish Ethical Review Act [35]. The participants were informed orally that their participation was optional, that they could withdraw from the study without giving a reason and that the answers were anonymous. Informed consent was considered given when the surveys were handed back.

### The survey

A study-specific survey was developed by the authors, who has competence in nursing education, telephone nursing, gender studies and survey development including psychometrics. The survey was pilot tested on a small group of university teachers in healthcare educations, and some alterations were thereafter made to enhance clarity. Furthermore, the survey had been used in several previous studies on nursing students and telephone nurses [16, 36, 37]. Because of the telephone nursing context, three aspects of intersectionality were chosen: age, ethnicity and sex, because telephone nurses mostly know these when a healthcare telephone call is made. The survey included descriptions of twelve fictive people who differed in age, ethnicity and sex: half of them with female-indicating names and half with male-indicating names; and described as either 25 years, 45 years and 70 years; and a native Swede or born outside Europe. The survey was made so that short descriptions of 2/12 fictive persons were used for each participating nurse teacher.

The items in the survey concerned estimations of the probability that, if the fictive person had called SHD or if s/he was referred to a physician when calling. Furthermore, the study participants were asked to assess whether the fictive person had a high quality of life, power over their own life and if they had had experiences of discrimination. The below described fictive persons used in the following study were:

“Isa” - female aged 25 years, Swedish origin

“Lynn” - female aged 25 years, non-European origin

“Johanna” - female aged 45 years, Swedish origin

“Manuela” - female aged 45 years, non-European origin

“Karin” - female aged 70 years, Swedish origin

“Li-Xing” - female aged 70 years, non-European origin

“Alexander” - male aged 25 years, Swedish origin

“Elliot” - male aged 25 years, non-European origin

“Björn” - male aged 45 years, Swedish origin

“Urghesa” - male aged 45 years, non-European origin

“David” - male aged 70 years, Swedish origin

“Ahmed” - male aged 70 years, non-European origin

First, each participant was randomly assigned two of the fictive persons and asked to estimate their likelihood of those persons had called SHD for help in healthcare related matters, if they had gotten a doctor’s appointment or if they had power over their own life. Free text comments could also be added. There were 66 combinations ([12 × 11]/2) to assess.

### Data analysis

For each assessed fictive person (“Isa” to “Ahmed”), the mean was calculated for each item. The estimations of each fictive person were then ranked from the lowest probability to the highest probability (12 ranked positions were possible). The reliability and homogeneity of the good-life index was calculated with Cronbach’s alpha coefficient (Cronbach, 1951) in the following way: (quality of life) + (power over own life) + (the reversed value of experience of discrimination)/3. The good-life index varied from 1 (very low) to 6 (very high). The nursing teachers experience of having called SHD depending on sex and age was calculated with Chi-square test. Significance level was set to 0.05.

## Results

The number of estimations for the 12 fictive persons is presented in Table 1.

**Table 1:** Number of assessments for the different fictitious persons (*n* = 135). Swedish-indicating names are presented in italics

Fictitious person	Number of assessments
*“Isa” female 25*	23
“Lynn” female 25	18
*“Johanna” female 45*	21
“Manuela” female 45	25
*“Karin” female 70*	24
“Li-Xing” female 70	22
*“Alexander” male 25*	25
“Elliot” male 25	22
*“Björn” male 45*	21
“Urghesa” male 45	17
*“David” man 70*	23
“Ahmed” male 70	26

### The estimation of having called SHD and being recommended doctor’s appointment

The probability of having called SHD was ranked as highest for the three fictive females of Swedish origin, and lowest for the three fictive males with non-European origin. The probability of having received a recommendation for a doctor’s appointment was highest for the two oldest fictive persons of Swedish origin and lowest for two fictive females of non-European origin. Please see Table 2.

**Table 2:** Assessment of probability of the fictitious persons having called SHD and receiving a recommendation of doctor’s appointment when calling. Swedish-indicating names are presented in italics

Probability of having called SHD				Probability of obtaining an appointment			
Rank	Fictitious person	Mean	SD	Rank	Fictitious person	Mean	SD
1	*“Johanna”*	4.76	1.1	1	*“David”*	3.78	1.0
2	*“Isa”*	4.52	1.3	2	*“Karin”*	3.71	1.0
3	*“Karin”*	4.33	1.1	3	“Ahmed”	3.50	1.3
4.5	“Manuela”	3.64	1.2	4	*“Björn”*	3.48	0.9
4.5	*“Alexander”*	3.64	1.4	5	*“Isa”*	3.43	1.1
6	*“Björn”*	3.62	1.1	6	*“Alexander”*	3.32	0.9
7	*“David”*	3.52	1.2	7	“Elliot”	3.27	1.2
8	“Urghesa”	3.47	0.7	8	“Lynn”	3.22	1.3
9	“Lynn”	3.17	1.3	9	“Urghesa”	3.18	1.1
10	“Elliot”	2.86	1.0	10	*“Johanna”*	3.05	1.2
11	“Ahmed”	2.69	1.2	11	“Li-Xing”	3.00	1.2
12	“Li-Xing”	2.18	1.0	12	“Manuela”	2.92	1.0

### The estimation of living a good life in Sweden

A good-life index was calculated, which consisted of the estimations of the fictive persons’ quality of life, power over their own lives and the reversed estimations of discrimination. The fictive persons assessed to be most likely to lead a good life were males of Swedish origin, followed by females of Swedish origin. The fictive persons assessed to be least likely to lead a good life were persons with non-European origin. Cronbach’s alpha for the index was 0.66 (Table 3).

**Table 3:** Good-life index, consisting of quality of life, power over one’s own life and the reversed assessment of experience of discrimination (range 3–18) for fictive persons. Rank, means and standard deviations (*n* = 135). Swedish-indicating names are presented in italics

Rank	Fictitious person	Mean	SD
1	*“Björn”*	12.81	1.5
2	*“Alexander”*	12.80	1.9
3	*“David”*	12.22	1.9
4	*“Johanna”*	12.10	1.8
5	*“Karin”*	11.70	2.4
6	*“Isa”*	11.00	2.4
7	“Lynn”	10.11	1.8
8	“Ahmed”	9.88	2.1
9	“Urghesa”	9.71	1.9
10	“Li-Xing”	9.45	2.1
11	“Manuela”	9.24	2.4
12	“Elliot”	9.14	2.2

### The estimation of having experiences of discrimination

Fictive persons with non-European origin were estimated to have the highest probability of experiencing discrimination. The fictive persons estimated to be least likely to have experienced discrimination were three males of Swedish origin, followed by females of Swedish origin. Please see Table 4.

**Table 4:** Experience of discrimination of fictive persons. The person who was expected to experience the most discrimination is ranked 1, and the least expected to experience it is ranked 12. Rank, means and SD are presented. Swedish-indicating names are presented in italics

Rank	Fictitious person	Mean	SD
1	“Urghesa”	4.53	0.9
2	“Manuela”	4.44	1.1
3	“Elliot”	4.36	1.2
4	“Ahmed”	4.35	1.1
5	“Lynn”	4.33	1.2
6	“Li-Xing”	4.23	1.0
7	*“Isa”*	3.61	1.6
8	*“Karin”*	3.21	1.2
9	*“Johanna”*	2.86	1.2
10	*“David”*	2.61	0.8
11	*“Björn”*	2.33	0.5
12	*“Alexander”*	2.28	0.7

### The nursing teachers’ experiences of calling SHD

Of the 135 nursing teachers, 110 had called SHD (81.5%), and 25 (18.5%) had not. Of those who had called, 105 were females and five were males. This indicates a significant difference (Chi-square = 4.295, degrees of freedom (df) = 1, *p* = 0.038). There was also a significant age difference: those who had called SHD were younger (M = 50.46 years, SD = 8.7) than those who had not called (M = 55.12 years, SD = 8.6, t = –2.389; df = 132; *p* = 0.018).

Because only nine out of 135 nurse teachers were men, it was not statistically possible to compare the assessments of the fictive persons and establish potential differences between males and females who responded to the survey.

## Discussion

According to our results, the nursing teachers were aware of inequity and discrimination in healthcare. They also expressed an awareness of the interaction between different power structures. The participants found it possible to assess the fictitious persons’ estimated life situation, probability of having a good life and access to SHD, even though the information provided was limited, as only age, ethnicity and sex of the fictitious persons were presented. Hence, the results of the present study support a previous study on nursing students’ awareness of equity in health [16]. Therefore, it could be concluded that both nursing teachers and nursing students in Sweden have a high awareness of intersectional aspects and of discrimination in healthcare. This might not be surprising, as Sweden has a longstanding reputation for equity in all areas of society. Further, such values echo in the Swedish Healthcare Act [4] and the Patient Act [5]. The study sample consisted of nursing teachers who were educated in nursing ethics, and they should therefore be aware of such aspects of care. There might, however, be a risk for social desirability bias, as the participants might be conscious about ‘the right values’ for a nursing teacher to express.

The context of the survey to the nursing teachers was telephone nursing. As the encounter in telephone nursing is “faceless”, it can be regarded as morally challenging [25]. This is in line with Levinas’ theory of encountering other persons as a face and thereby become aware of the moral demand the encounter raises [27]. However, the results of the present study indicated that another person could be imagined as “a face” also in a faceless encounter. This was shown by the respondents’ ability to estimate the life situation and quality of life of the fictitious persons, although the only information they had were their sex, age and ethnicity. Likewise, the participants’ awareness of (in)equity in care can be interpreted as an expression of seeing other persons as faces, that is, as moral persons with the right to dignity and equal treatment. These are important values which should permeate nursing education.

To be aware of a phenomenon is, however, one thing and to behave according to best practice is another. The present results do not tell if the nursing teachers thought these issues were important or whether they permeated their teaching. Awareness could, at its best, be a step in the chain towards actions, as previously described [37]. There could, however, be a fine line between awareness and prejudice. Gender-related issues have been described as important but of low priority by medical teachers [38]. Similar studies of nursing teachers were not found, why this is an area that calls for further investigation. Andersson et al. [39], in their study of medical students’ awareness of gender in medicine, suggested that to raise interest among students about gender aspects in health and healthcare and make them aware of the importance of gender, it is pivotal that the examples used in discussions are trustworthy and relevant. Furthermore, it is important to create a climate for dialogue in which students feel permitted to share ideas and perceptions to create an awareness [39]. As long as female patients are less likely to get expensive medicines and have to wait longer for GP appointments [10], there is need to raise awareness within healthcare. There might also be discrimination because of age or ethnicity, and there is an additional need to raise awareness about sensitivity to different groups of patients [21].

Moreover, Sweden, like several other countries, currently encounter swift cultural change and growing demands for increased plurality within nursing to recruit and retain minority groups in the workforce [40]. Flood and Commendador [15] underscored that transcultural nursing can be learned, but there is room for reforms. We argue that registered nurses and nursing students should understand intersectionality and cultural aspects and act with sensitivity in patient encounters. From the present findings, it can be concluded that nursing teachers consider males to possess more power than females, but that ethnicity is the single factor that seems to affect their estimations the most. Programs to increase cultural competence have shown promising results [14]. Such programs could be further developed to embrace a broader and intersectionality perspective. We suggest activities to raise the awareness of the importance of these aspects, in accordance with Risberg et al. [38] suggestions. The present survey could be used for such work. Tengelin & Dahlborg-Lyckhage [41] also point to the importance of incorporating a norm-critical perspective in nursing education.

Concerning knowledge and use of the service SHD 1177, the results showed significant differences between females and males. More females than males had called SHD and those who had used the service were also younger in age. These results are in line with the findings of the study on nursing students, using the same survey.

### Strengths and limitations

The survey in the present study was formulated in the context of telephone nursing, aiming to reflect discrimination and equity in connection to calling SHD. That the study was small scaled and conducted in a Swedish setting, are obvious limitations. A further limitation is the convenience sample. About two thirds of nursing teachers at the three universities answered the survey, which is an acceptable response rate. As is common in nursing education, the sample of nursing teachers included few males. Cronbach’s alpha for the good-life index was 0.66, which is slightly lower than the desired value of 0.70 [42].

## Conclusions

The present study indicated that the nursing teachers were aware of how age, ethnicity and sex are related. This means, that they were aware of discrimination and inequity in healthcare. This is important, as their values are pivotal in education. The participants were able to estimate a fictive person’s life situation based on the limited knowledge of age, ethnicity and sex. The created good-life index showed that the nursing teachers rated younger male persons to lead the best lives. Eventually, all nursing teachers knew of SHD, but more female teachers than male teachers had used the service. The present survey could be used as a starting point for a discussion of equity in healthcare. It could also be used for interventions aiming at increasing awareness of discrimination and inequity.

## Supplementary Information

**Additional file 1.**

## Data Availability

The datasets generated and/or analysed during the current study are not publicly available due to ethical reasons and the right to confidentiality for recorded persons but are available from the corresponding author on reasonable request.

## Abbreviations

ICN: International Council of Nurses
SHD: Swedish Healthcare Direct
UN: United Nations
