# Supplementary material for: A survey of nursing teachers’ awareness of discrimination and inequity in telephone nursing care
Source: BMC Nurs. 2021 Dec 2;20:240. doi: 10.1186/s12912-021-00762-5 (PMC8638536; doi:10.1186/s12912-021-00762-5)
Supplement: Supplementary file 1 — Additional file 1. [file 12912_2021_762_MOESM1_ESM.docx]

**Your task is to assess how two fictive persons could possibly experience the situations described. There is no right or wrong answer. The intention is to shed light on how different aspects could impact on how persons are treated and experience living in today’s society.**

First some questions about you:

1. Gender female ( )

male ( )

other ( )

1. Year of birth ?
2. What is your professional title?

lecturer ( )

senior lecturer ( )

other ( )

1. Country of birth?

1. Have you ever called Swedish Healthcare Direct for care and advice?

Yes ( )

No ( )

**Karin is 70 years and native Swede**

1. **Is she likely to have called Swedish Healthcare Direct?**

Not likely at all ( )

Probably not ( )

Somewhat likely ( )

Rather likely ( )

Very likely ( )

Very high probability ( )

1. **Is it likely that she will get a doctor’s appointment if she calls Swedish Healthcare Direct?**

Not likely at all ( )

Probably not ( )

Somewhat likely ( )

Rather likely ( )

Very likely ( )

Very high probability ( )

1. **Is it likley that she leads a good life with high quality of life?**

Not likely at all ( )

Probably not ( )

Somewhat likely ( )

Rather likely ( )

Very likely ( )

Very high probability ( )

1. **Is it likely that she experiences power over her own life??**

Not likely at all ( )

Probably not ( )

Somewhat likely ( )

Rather likely ( )

Very likely ( )

Very high probability ( )

1. **Is it likely that she lives alone?**

Not likely at all ( )

Probably not ( )

Somewhat likely ( )

Rather likely ( )

Very likely ( )

Very high probability ( )

1. **Is it likely that she suffers from substance abuse?**

Not likely at all ( )

Probably not ( )

Somewhat likely ( )

Rather likely ( )

Very likely ( )

Very high probability ( )

1. **Is it likely that she is working?**

Not likely at all ( )

Probably not ( )

Somewhat likely ( )

Rather likely ( )

Very likely ( )

Very high probability ( )

1. **Is it likely that she has experienced discrimination?**

Not likely at all ( )

Probably not ( )

Somewhat likely ( )

Rather likely ( )

Very likely ( )

Very high probability ( )

1. **Is it likely that she has been on longterm sickleave?**

Not likely at all ( )

Probably not ( )

Somewhat likely ( )

Rather likely ( )

Very likely ( )

Very high probability (

Please feel free to comment below:
